# 2D and 3D mapping of traffic induced noise near major roads passing through densely populated residential area of South Delhi, India

**DOI:** 10.1371/journal.pone.0248939

**Published:** 2021-03-24

**Authors:** Pervez Alam, Kafeel Ahmad, Afzal Husain Khan, Nadeem A. Khan, Mohammad Hadi Dehghani

**Affiliations:** 1 Department of Civil Engineering, Baba Ghulam Shah Badshah University, Jammu and Kashmir, India; 2 Department of Civil Engineering, Jamia Millia Islamia, Central University, New Delhi, India; 3 Department of Civil Engineering, Jazan University, Jazan, Saudi Arabia; 4 Department of Environmental Health Engineering, School of Public Health, Tehran University of Medical Sciences, Tehran, Iran; 5 Institute for Environmental Research, Center for Solid Waste Research, Tehran University of Medical Sciences, Tehran, Iran; Al Mansour University College-Baghdad-Iraq, IRAQ

## Abstract

Noise monitoring and mapping is the critical processes to ensure that the noise level does not reach the harmful levels and provides noise exposure level details. 2-D and 3-D noise mapping has been carried out at pre-selected critical locations of major roads passing through densely populated residential areas, namely, Mathura Road, Lodhi Road, Lala Lajpat Rai Road, and Ring road, along with significant intersections, viz. Moolchand, Ashram, Sabz Burj, and Lodhi road. The monitoring has been performed during the day and night’s peak traffic hours using Sound Level Meter (SLM) Larson & Davis 831as per standard procedure. Then after, 2-D and 3-D noise maps have been prepared, visualized, and analyzed by soundPLAN (acoustic) and MapInfo Pro (Desktop GIS). The maximum noise level is observed at Ashram Chowk [81.1 dB (A)] at 8 pm; however, the minimum noise level is found to be at Lala Lajpat Rai Road [76.4dB (A)] at 7 pm. Monitoring results of noise level show non-compliance of regulatory standards for day time and night time. 2-D noise maps revealed that the noise level is maximum at the centerline of the road and decreases either side with the distance, and remains above the permissible limits at all locations. However, the 3-D noise maps show horizontal as well as vertical noise levels at all locations. The 3-D noise maps also revealed a noise level of 70 dB (A) up to a height of 6.096m at the Ashram Chowk and Moolchand intersection. However, a noise level of 65 dB (A) has been observed at the height of 5.486m at Lala Lajpat Rai Marg and Sabz Burj. This study will explore noise levels in both horizontal and vertical directions near roads surrounded by high-rise buildings. It will help the decision-makers take remedial measures.

## Introduction

In recent times, noise pollution has been recognized as one of the major apprehensions that adversely impact the quality of life in urban areas across the globe [1]. With the hasty increase in urbanization, industrialization, and other communication of transport systems, noise pollution has reached a distressing level over the years [2]. Before suggesting mitigative measures, it is imperative to identify the source/sources of noise, its levels, and critical locations where immediate remedial measures are required. Noise monitoring plays a vital role in finding critical noise exposure locations [3, 4]. It helps to identify work locations where there are noise problems, people who may be affected, and where additional noise measurements need to be made.

Noise mapping covers the entire mapping process from the collection of data, the storage and retrieval of this data for modeling/computation to demonstrate information related to outdoor sound levels, sound exposure, noise effects, or numbers of affected persons [5, 6]. Noise mapping is one of the best ways to understand environmental noise levels, origin, sources, and distribution [7, 8]. Noise maps are the graphical presentation of noise levels according to different color combinations [9, 10]. Contour lines are used to show the boundary of varying noise levels in a particular area. Noise maps are also helpful in establishing the existing baseline data to measure the effectiveness of future initiatives to control noise pollution [11]. It may use in understanding and visualizing the way noise spread from the roads to residential areas and helps to see the effects of noise due to nearby buildings along the roads’ periphery.

In India, the environmentalists have raised their severe concern and resolved to move towards pressing noise mapping methodology to identify and quantify the scale of noise affecting the human population. Akhter et al., 2002 [12] revealed that the noise level of selected locations of Delhi had been observed in the range of 53 dB (A) to 83 dB (A). Many researchers have revealed that noise mapping is one of the most effective and reliable tools for analyzing noise in an urban environment [13–17]. Noise mapping ultimately gives a lucid image of traffic movements at the specific locality and becomes helpful to traffic management [18, 19]. Merchan and Diaz, 2013 [20] reported that noise mapping is an economic process that could save them time and money for decision-makers to take remedial measures. Joshi et al., 2015 [21] observed through noise maps of Mumbai city that most of the areas had been exposed with a very high intensity of noise range from 63.8dB (A) to 80.2 dB (A). In another study conducted by Pinto and Mardones, 2008 [22] on Noise-mapping of Copacabana, Rio-de-Janeiro (Brazil) shows that noise maps are a perfect approach to noise level distribution of any area. Billah and Rahman, 2004 [23] developed noise maps for the Sahbagh area of Dhaka city, Bangladesh, to know the real noise pollution situation. Results of noise maps point out the higher sound pressure level during working days than in the holidays because of high traffic volume. The noise maps have been prepared for Santiago, and Chile results revealed that the noise levels were very high Suarez and Barros, 2011 [24]. Geymen and Bostanci, 2012 [25] carried out their study to develop GIS aided noise maps using the Geostatistical Analyst module on arc GIS. They found that noise was generally exceeding the permissible limit, which is regarded as unsafe. Noise Mapping also helped urban planners to reduce the irritation experienced by big-city residents, such as road traffic noise and industrial noise Gonzalez et al., 2015 [26]. In another study done by Alam et al., 2019 [27] for city Delhi they investigate the temporal and spatial distribution of noise level in the closed vicinity of urban roads. Literature review shows that near peripheral roads of urban settings, 2-D and 3-D noise mapping studies are lacking. Therefore, the present research focused on 2-D and 3-D noise mapping at pre-selected critical locations of major roads passing through densely populated residential areas, namely, Mathura Road, Lodhi Road, Lala Lajpat Rai Road, and Ring road along with significant intersections, viz. Moolchand, Ashram, Sabz Burj, and Lodhi road.

## Methodology

### Study area

**Fig 1:**
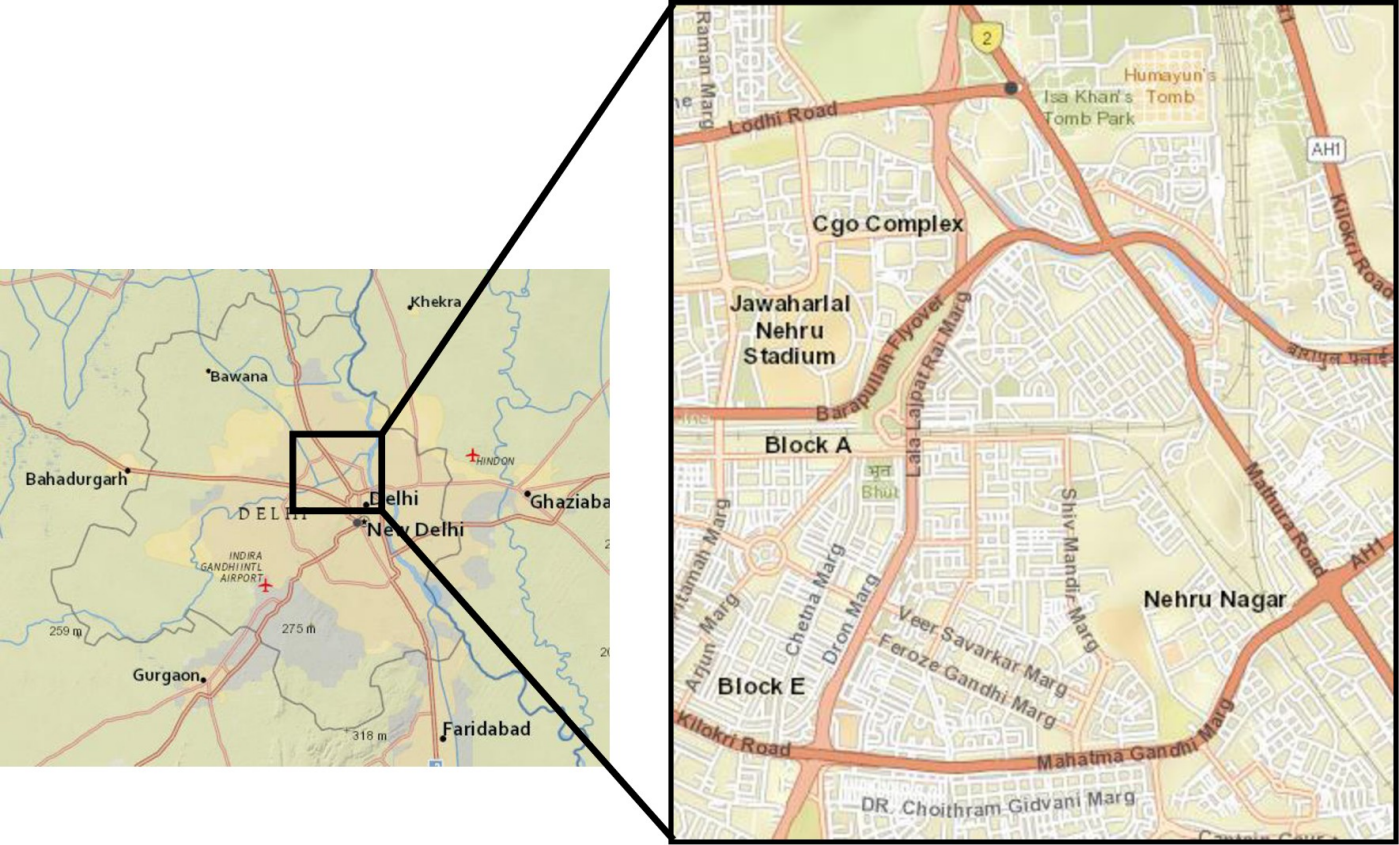
Geographical location of New Delhi (India) and study area (Satellite imagery source: The National Map; http://viewer.nationalmap.gov/viewer/).

Delhi is situated in northern India between the latitudes of 28°-24’-17" and 28°-53’-00" North and longitudes of 76°-50’-24" and 77°-20’-37" East. The city is divided into eleven revenue districts and has a population of 18.6 million. Delhi is the most populous city in India; that’s why a residential area has been selected for the study. Fig 1 shows the geographical location of Delhi and the study area.

### Noise monitoring station

**Fig 2:**
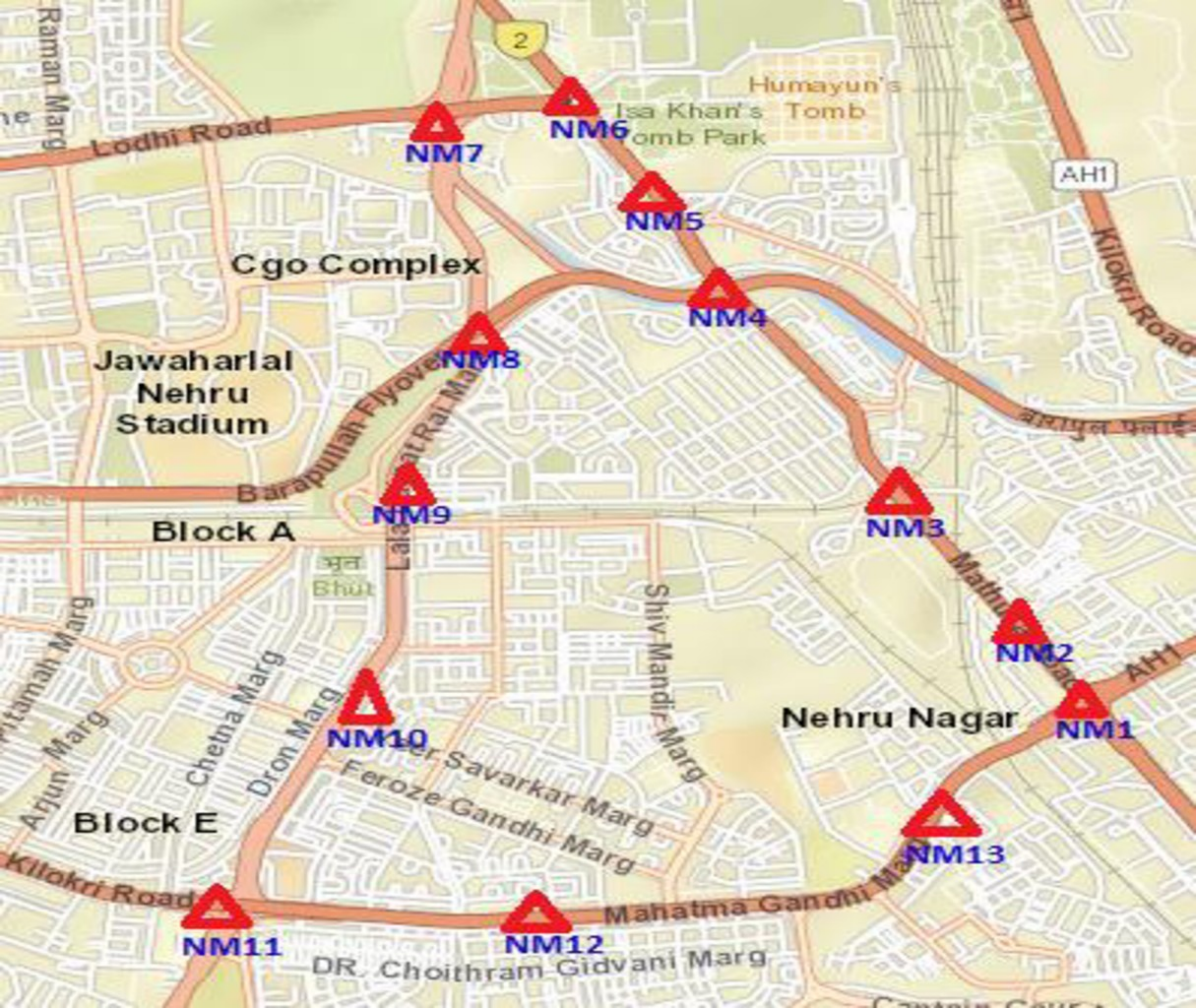
Spatial distribution of noise monitoring station on satellite imagery of Delhi City, India. The red triangle symbol indicates noise monitoring stations. (Satellite imagery source: The National Map; http://viewer.nationalmap.gov/viewer/).

Thirteen temporary noise monitoring stations, i.e., Noise monitoring Station (NM1) to Noise monitoring station (NM 13) shown in Fig 2 and Table 1, have been established for 24 hr noise monitoring. To select the noise monitoring station, special care has been made to cope with the different road conditions of the study area. All cross sections of the study area have been covered for noise monitoring. Noise monitoring has been done in good weather conditions to reduce the effects of wind and rain. Wind shield has been used to reduce the effect of wind and rain.

**Table 1:** Description of noise monitoring stations.

S. No	Site ID	Name of site	GPS Coordinate of sampling locations	
			X (Easting)	Y (Northing)
1	NM1	Ashram Chowk	77°34’30”	28°34’21.1”
2	NM 2	Ashram Bus stand (Hari Nagar)	77°25’63”	28°57’48”
3	NM 3	Jangpura	77°25’09”	28°58’33”
4	NM 4	Bhogal	77°24’96”	28°58’46”
5	NM 5	Nizammuddin	77°24’51”	28°59’11”
6	NM 6	Sabz Burg	77°24’33”	28°59’30”
7	NM 7	Lala Lajpat Rai Road (Below Fly over)	77°23’99”	28°59’27”
8	NM 8	Lala Lajpat Rai Road (Near HP Petrol 10 pump)	77°24’08”	28°58’58”
9	NM 9	Defence Colony Flyover (Near Block C)	77°24’08”	28°58’58”
10	NM 10	Lajpat Nagar II Defence Colony	77°23’61”	28°57’03”
11	NM 11	Below Moolchand Flyover	77°23’41”	28°56’53”
12	NM12	Lala Lajpat Rai Marg (Near IBS Hospital)	77°24’43”	28°56’52”
13	NM 13	Mahatma Gandhi Road (Nehru Nagar below Foot over Bridge)	77°25’38”	28°56’86”

### Land use zoning of study area

**Fig 3:**
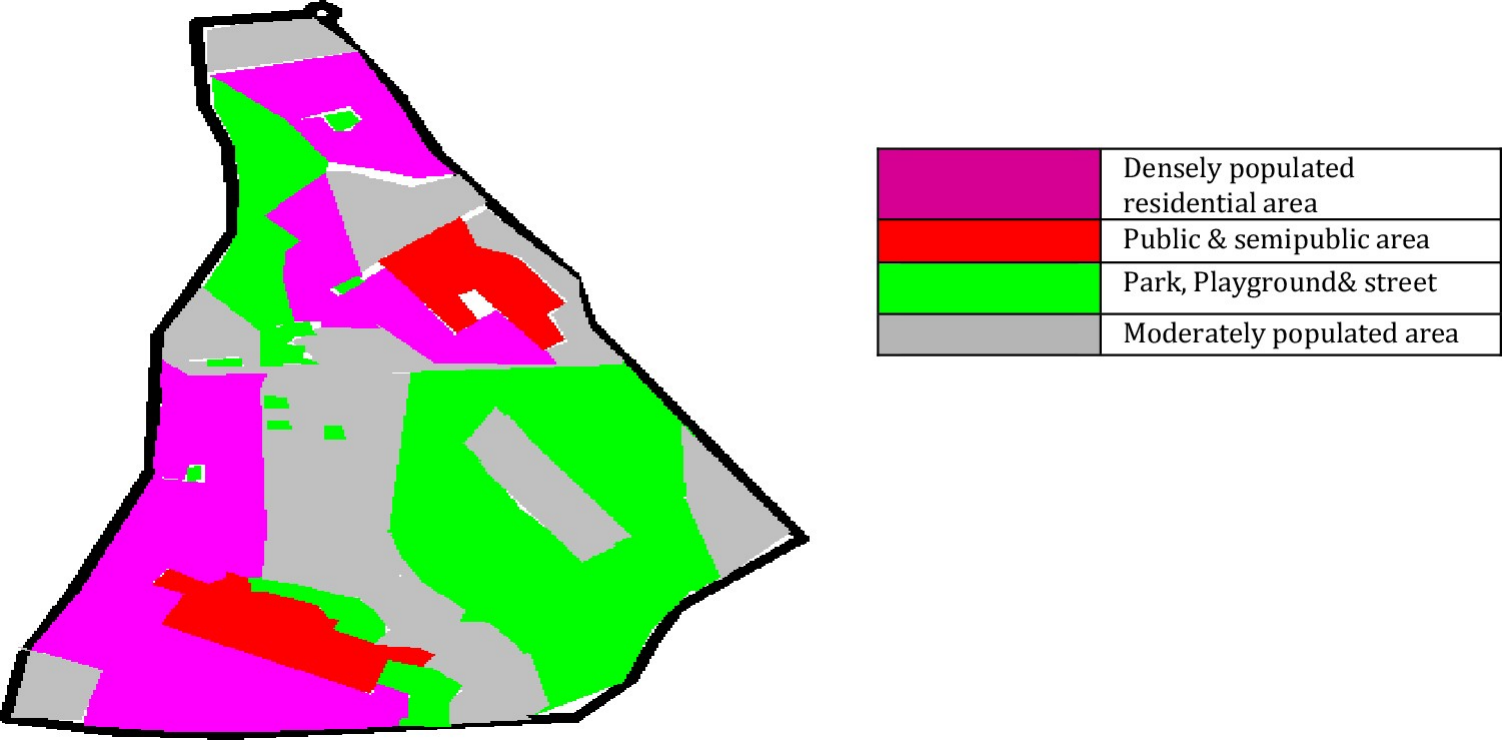
Spatial distribution of land use zones of study area. The colors of the area correspond to different land use zones.Pink: Densely populated residential area;Red: Public and semipublic area; Green: Park, playground and street; Grey: Moderately populated area. A version of this figure with the original imagery is available from the authors.

The study area currently has four land use zones “(1) Densely populated residential area (2) Public and semipublic area (3) Park, playground and street (4) Moderately populated area”. Fig 3 shows the distribution of land-use patterns of the study area. It shows that 30% of the study area is densely populated, 14% is Public and semipublic area, 26% Park, playground, street, and the rest 30% is moderately populated. The regulatory organization Central Pollution Control Board (CPCB), India has divided any city or area into four zones, i.e., Residential, Commercial, Industrial, and Silent zones, and prescribed ambient noise standards (Day time and Night time) for respective zone.

### Instrumentation

**Fig 4:**
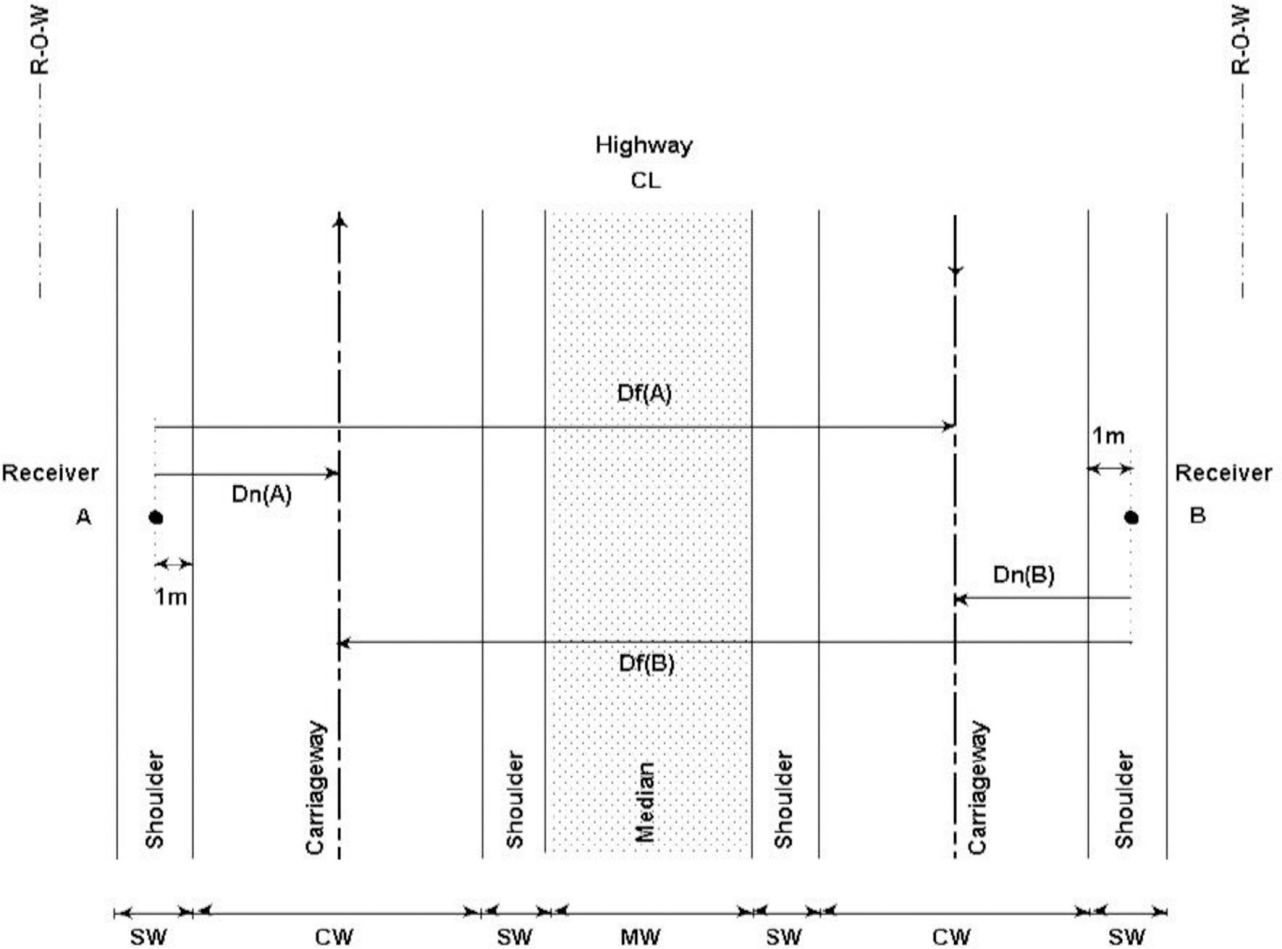
Position of SLM from the median and end of the road.

In Delhi, it has been observed that the traffic density in the morning remains more at one side of the road, and density reverses in the evening. Fig 4 shows a four-lane city road; a four-lane highway is generally kept with a two-carriage way (CW) for both sides of the traffic movement. Median way (MW) acts typically as the center of the road, and shoulder way (SW) is mainly provided along both side of the road and some time with the median. Two sound level meters Sound Level Meter (SLM) Larson & Davis 831 have been used at both sides of the road at 1 m away from the road end. The average noise level of the two SLM has been used for noise mapping and analysis. According to Akhter et al., 2010 [28], the microphone location also influence the results of urban noise measurements.

### Noise monitoring procedure

The ambient noise monitoring in Leq(A) is found to be suitable as per Central Pollution Control Board (CPCB, 2005) [29]. Later, the same has been confirmed by ISO 1996–1 in 2016 [30] and recognition as an international standard. Therefore, Leq(A) noise level has been adopted to monitor and analyze the environmental noise level. The noise monitoring has been done as per the standard method recommended by the Central Pollution Control Board CPCB, 2005 and suggested by ISO 1996 part 1 and part 2. 2D and 3D noise maps of critical locations have been developed to investigate the existing distribution of noise in these locations.The following standard procedure has been adopted for noise monitoring:

- Tripod stand has been kept above the ground level of 1.5 m.
- Environment conditions: low relative humidity and low wind speed (less than 5 m/s).
- The instrument has been calibrated at every noise monitoring station.
- Noise monitoring has been done on working days only.
- Furthermore, special care has been taken to reduce the effect of wind.

As prescribed in the Table 2, 24 hr noise monitoring has been conducted from 18th of July to 12th of August, 2016 as per standard procedure and result has been analyzed.

**Table 2:** Noise monitoring location, date and time.

S. No	Site ID	Monitoring Date	Monitoring Time Period	
			Day Time	Night Time
1	NM1	18/07/2016	6 AM to 10 PM	10 PM t 6 AM
2	NM 2	20/07/2016	6 AM to 10 PM	10 PM t 6 AM
3	NM 3	21/07/2016	6 AM to 10 PM	10 PM t 6 AM
4	NM 4	24/07/2016	6 AM to 10 PM	10 PM t 6 AM
5	NM 5	26/07/2016	6 AM to 10 PM	10 PM t 6 AM
6	NM 6	27/07/2016	6 AM to 10 PM	10 PM t 6 AM
7	NM 7	28/07/2016	6 AM to 10 PM	10 PM t 6 AM
8	NM 8	31/07/2016	6 AM to 10 PM	10 PM t 6 AM
9	NM 9	03/08/2016	6 AM to 10 PM	10 PM t 6 AM
10	NM 10	07/08/2016	6 AM to 10 PM	10 PM t 6 AM
11	NM 11	09/08/2016	6 AM to 10 PM	10 PM t 6 AM
12	NM 12	10/08/2016	6 AM to 10 PM	10 PM t 6 AM
13	NM 13	12/08/2016	6 AM to 10 PM	10 PM t 6 AM

### Noise mapping

Noise data has been collected for 13 locations of a residential area. After analysis, the maximum noise level had been found at four intersections, i.e., Ashram Chowk, Sabz Burj, Lala Lajpat Rai Marg, and Moolchand. Therefore, noise maps have been prepared only for these critical locations using SoundPLAN and Mapinfo (Desktop GIS).

### Software used

The noise monitoring results have been visualized and analyzed by the following software.

1. SoundPLAN (acoustic)
2. MapInfo Pro (Desktop GIS)

Digitalized maps are required for noise mapping that is fulfilled by MapInfo Pro. Noise level and digitalized maps have been used for the development of noise maps using SoundPLAN (acoustic).

### Noise mapping procedure

The following steps have been adopted for the development of noise maps of the pre-selected area.

- Digitalization of location map.
- Demarcation of noise monitoring station on the digitalized map.
- 24 hr noise monitoring data in excel sheet as per the noise monitoring station marked.
- Analysis of noise monitoring data and digitalized map for prediction.
- Development of noise maps using predicted data and digitalized maps using soundPLAN.

## Results and discussion

### Noise monitoring

**Fig 5:**
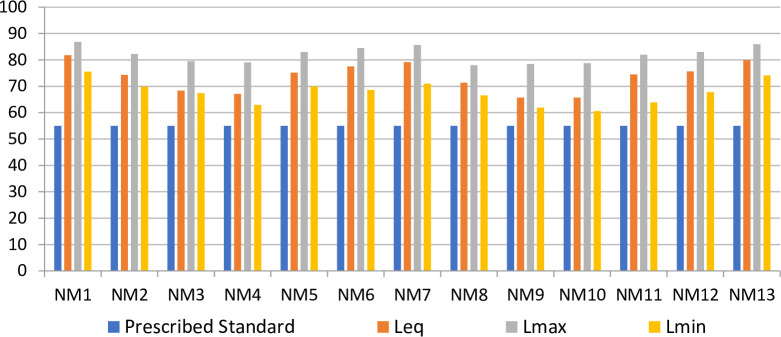
Variation of maximum, equivalent and minimum noise level at noise monitoring site in day time.

**Fig 6:**
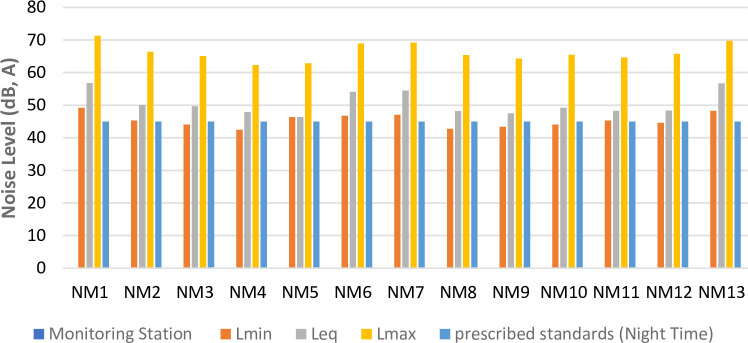
Variation of maximum, equivalent and minimum noise level at noise monitoring site in night time.

The maximum, equivalent and minimum noise level in the day time and night time is shown in Figs 5 and 6, respectively. The results revealed that the noise level exceeds the prescribed acceptable standards for daytime and night time. The maximum value of Leq, 81.8dB (A) has been found at Ashram Chowk, followed by 79.9dB (A) at Moolchand, 79.1dB (A) at Lala Lajpat Rai Road, and Sabz Burj 78.3dB (A). These four locations have been observed to be critical in terms of noise monitored data and heavy traffic volume. The results also show that the noise level at noise monitoring stations NM3, NM4, NM9, and NM10 is safe at night because of significantly less traffic volume.

### Discussion on noise maps

The area with the higher noise level, i.e., Ashram Chowk, Sabz Burj, Lodhi Road Cross Section, and Moolchand has been selected for 2D and 3D noise mapping as these locations are critical in terms of noise and traffic volume.

### Ashram Chowk

**Fig 7:**
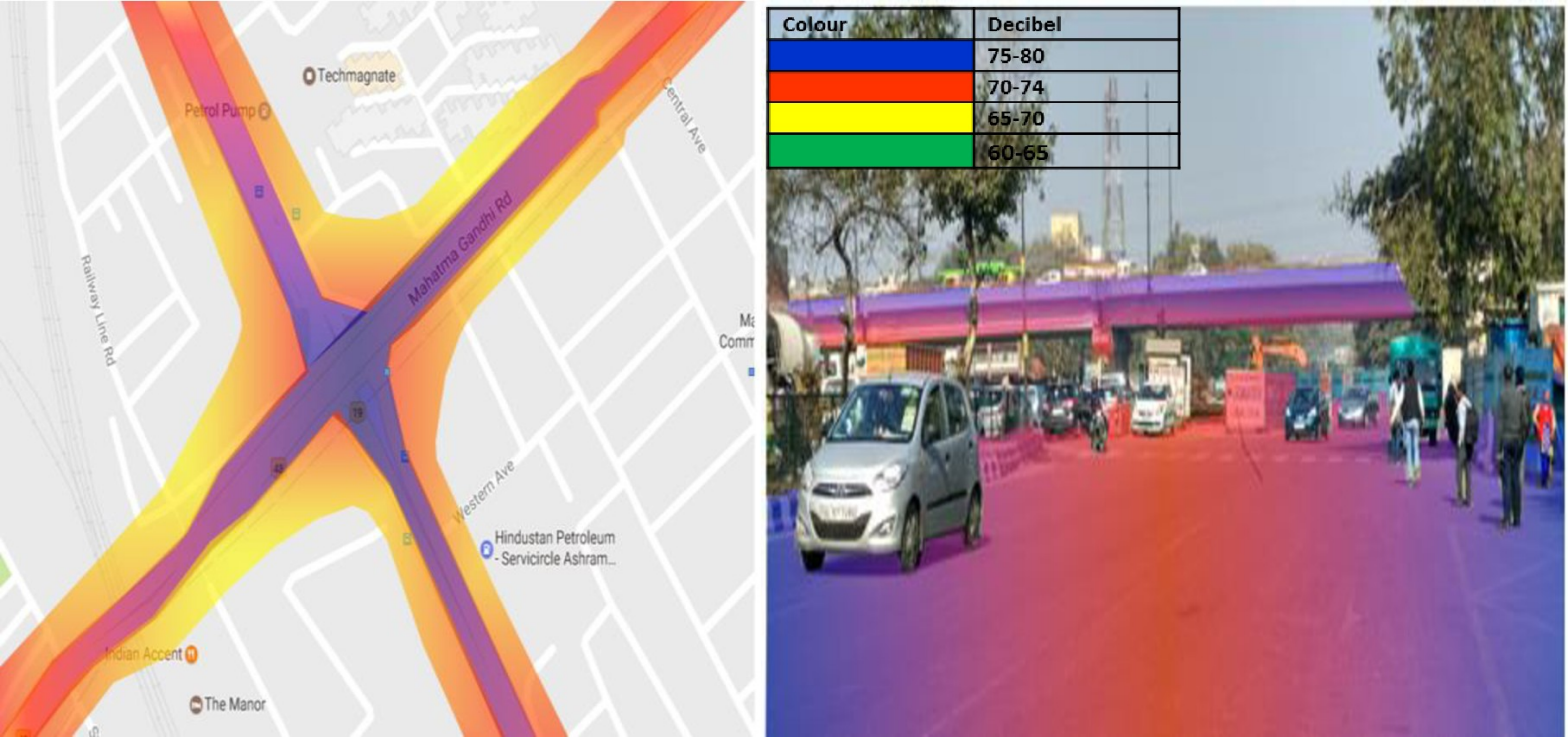
2D & 3D Noise mapping of Ashram chowk. The colors indicates the different noise zones.Blue: maximum noise level 75-80dB (A); Red: maximum noise level 70-74dB (A); Yellow:maximum noise level 65-70dB (A); Green: maximum noise level 60-65dB (A).A version of this figure with the original imagery is available from the authors.

2D and 3D noise map of Ashram Chowk has been shown in Fig 7. The maximum noise level, 75-80dB (A) (Colour Code-**Blue**), has been observed at Ashram Chowk in peak hours. This may be due to eco produced by multiple obstructions like flyover, intersection, and nearby high rise buildings. Furthermore, the noise level decreased with distance and has been observed to be 70–74 dB (A) (Colour Code-**Red**) at 46.5m from the road’s median. Additionally, it reaches upto 65–70 dB (A) (Colour Code-**Yellow**) at a distance of 63.5m from the median. However, noise level around the intersection has been found to be in the range of 70–74 dB (A) (Colour Code-**Red**) upto a distance of 83.5m. This may be due to high traffic volume and the addition of reflective noise. The Leq obtained from the 3D map revealed that the flyover that passes over the intersection of Ashram Chowk receives a noise level of 75–80 dB (A) (Colour Code- **Blue**) up to a height of 6.96m. This may be because of the high traffic volume and honking noise at the intersection.

Additionally, reflective noise due to flyover at the intersection is also added to the overall noise of Ashram Chowk. However, the noise level at the road’s median is observed to be 70–74 dB(A) (Colour Code-**Red**), less than that on the shoulder. This may be due to the reflection of noise from the shoulder and boundary wall. The 3D noise maps also show that nearby obstructions such as boundary walls and flyovers affect noise levels’ distribution pattern.

### Sabz Burj

**Fig 8:**
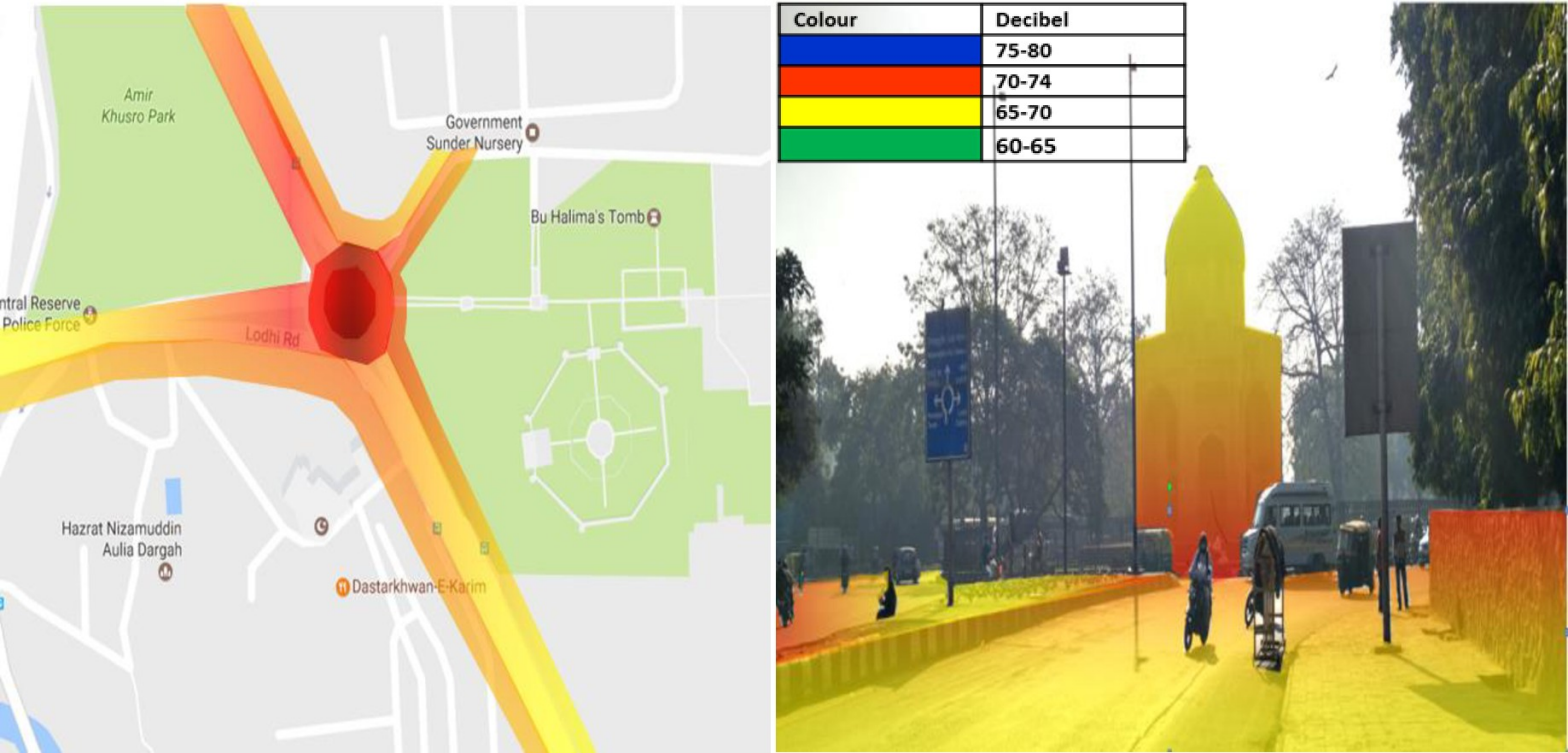
2D & 3D Noise mapping of Sabz Burj. The colors indicates the different noise zones.Blue: maximum noise level 75-80dB (A); Red: maximum noise level 70-74dB (A); Yellow: maximum noise level 65-70dB (A); Green: maximum noise level 60-65dB (A).A version of this figure with the original imagery is available from the authors.

The maximum noise level at Sabz Burj has been found to be in the range of 70–74 dB (A) (Colour Code-**Red**) at the roundabout of Sabz Burj shown in Fig 8. This may be because Sabz Burj is a roundabout, traffic flows from all directions, and drivers rapidly use horn due to less sight distance. 2D noise map also reveals that the noise level at the median of the road observed to be 65–70 dB (A) (Colour Code-**Yellow**), it reduces as we move away from the median and reaches up to the permissible limit at a distance of 98m from the median. Whereas 3D noise map reveals that the noise level is found to be in the range of 70–74 dB (A) (Colour Code- **Red**) up to a height of 4.65m at Sabz Burj, it decreases with height and reaches up to 60–65 dB (A)(Colour Code- **yellow**) at the height of 7.32m. It has also been observed from the 3D noise map that the boundary wall and shoulder of the road is experiencing a noise level of 70–74 dB (A) (Colour Code- **Red**). The median noise level is observed to be slightly less and found to be in the range of (Colour Code- **yellow**). This may be due to the addition of reflective noise due to shoulders and boundary walls. Although, results show that the noise level at Sabz Burj remains less than that of Ashram Chowk.

### Lodhi road

**Fig 9:**
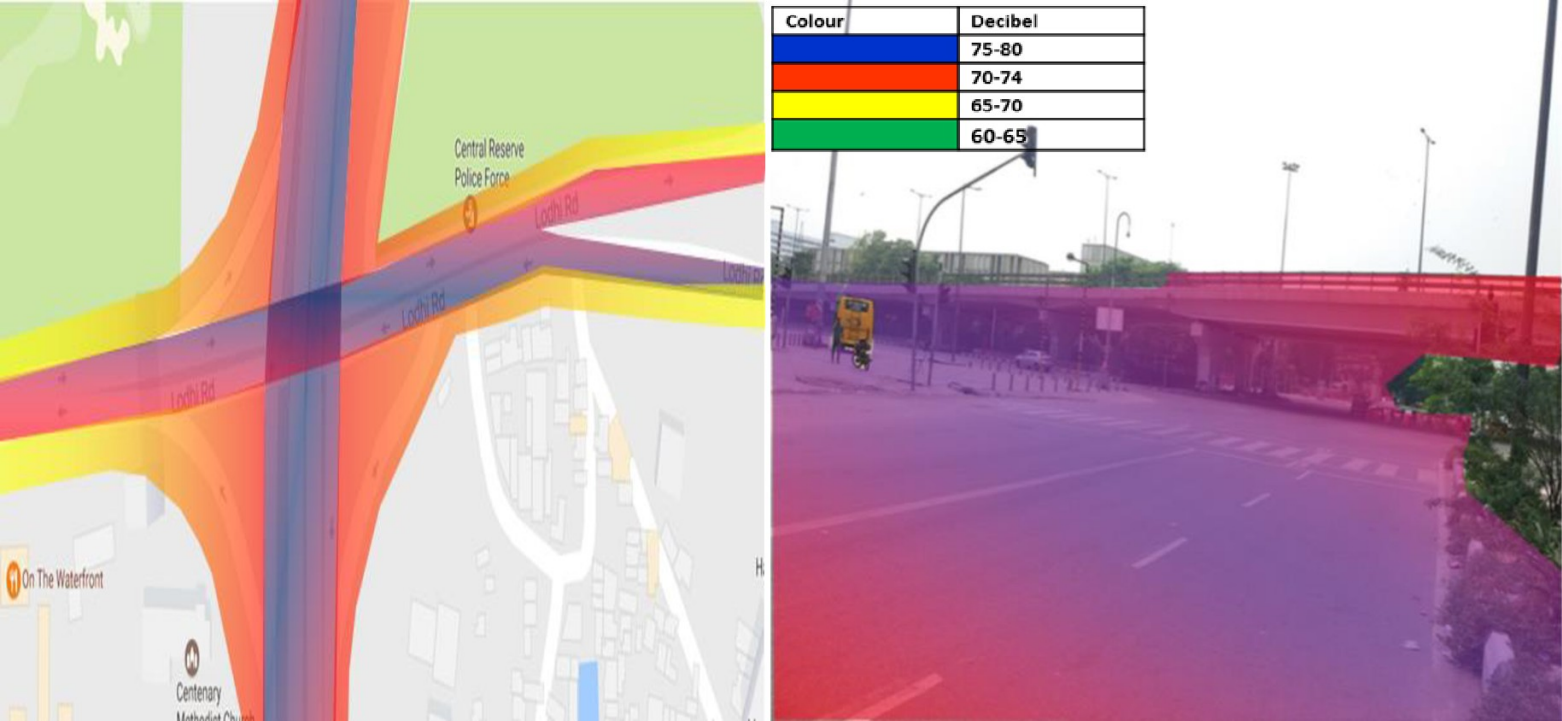
2D & 3D Noise mapping of Loadhi road. The colors indicates the different noise zones.Blue: maximum noise level 75-80dB (A); Red: maximum noise level 70-74dB (A); Yellow: maximum noise level 65-70dB (A); Green: maximum noise level 60-65dB (A). A version of this figure with the original imagery is available from the authors.

2D noise map of Lodhi road shows that the noise level at the median of the road remains maximum and found to be in the range of 75–80 dB (A) (Colour Code-**Blue**). As shown in Fig 9 the Lodhi Road’s high noise level may be due to intersection and reflective noise, as a flyover is passing over the selected road. Furthermore, it decreased on both sides of the road with the increase in distance and has been observed to be 70–74 dB(A) (Colour Code-**Red**) at a distance of 189.4m from the median, which is still more than the prescribed standard of CPCB. Additionally, the noise level is found to be in the range of 65–70 dB(A) (Colour Code-**Yellow**), at a distance of 396.4m from the center of the intersection. The 3D noise map revealed that the flyover passing over Lodhi Road’s intersection is exposed to a noise level of 70–74 dB (A) (Color Code-**Blue**) up to a height of 6.8 m. Noise level decreased on either side of the intersection and has been observed to be 70–74 dB (A) (Color Code-**Red**) at the same height. It has also been observed that the noise level reduces with distance from the intersection because less reflective noise reaches upto a distance of 205m from the intersection. Similar findings have been reported by Munzel.et. al.,(2017) [31], the nearby residential building of the study area has been exposed to a noise level of 70–80 dB upto a height of 8.5m and 60–70 dB upto a height of 12.5m.

### Moolchand cross section

**Fig 10:**
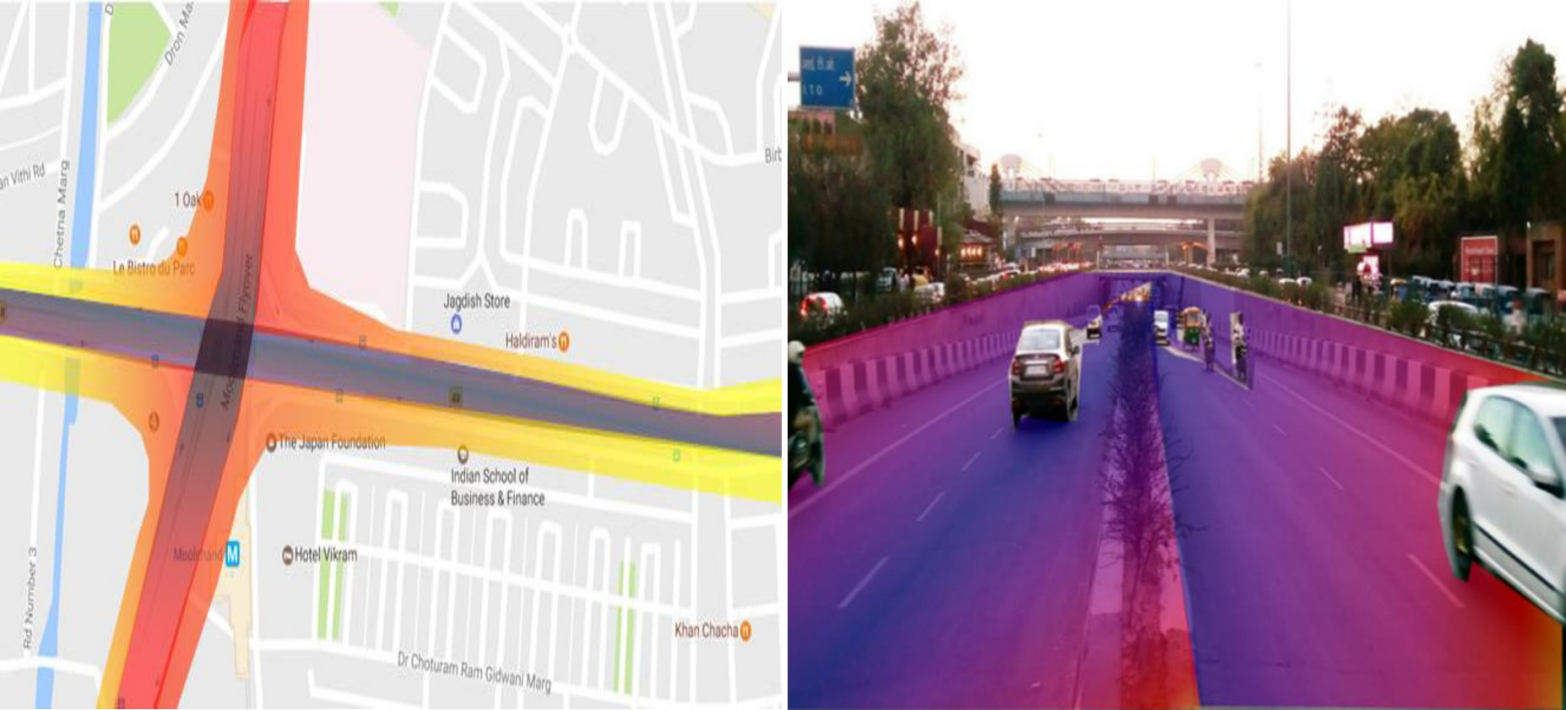
2D & 3D Noise mapping of Lodhi road. The colors indicates the different noise zones.Blue: maximum noise level 75-80dB (A); Red: maximum noise level 70-74dB (A); Yellow: maximum noise level 65-70dB (A); Green: maximum noise level 60-65dB (A). A version of this figure with the original imagery is available from the authors.

The 2D and 3D noise map of the Moolchand Cross Section is shown in Fig 10. The maximum noise level has been observed at the intersection and found to be 75-80dB (A) (Colour Code-**Blue**). This may be due to echo produced by multiple obstructions like Flyover, the underpass, and an elevated metro rail line simultaneously. Furthermore, intersection traffic always remains very high, and the metro also contributes to the overall noise. The noise level reaches upto 65–70 dB(A) (Colour Code-**Yellow**), at a distance of 201.8 m. However, the noise level is observed to be in the range of 70–74 dB(A) (Colour Code-**Red**) towards Lala Lajpat Rai Marg upto a distance of 205m from the center of the intersection because of high rise building on both sides. It can be observed from the Fig 10 that the noise level at the junction has been continuously more than 80dB (A)(Color Code-**Blue**) up to a height of 3.05m; it decreases with height and reaches 65 dB (A)(Color Code-**Green**) at the height of 6.19m. 3D noise map also shows that the nearby Moolchand hospital is also exposed to a high noise level of 56 dB (A) up to a height of 9.34m. Since Moolchand Hospital comes under the silent zone, a noise barrier of noise reduction coefficient 0.9 with height of 2.5m will be beneficial to reduce the noise level up to the desired standard.

## Validation of noise mapping results

**Fig 11:**
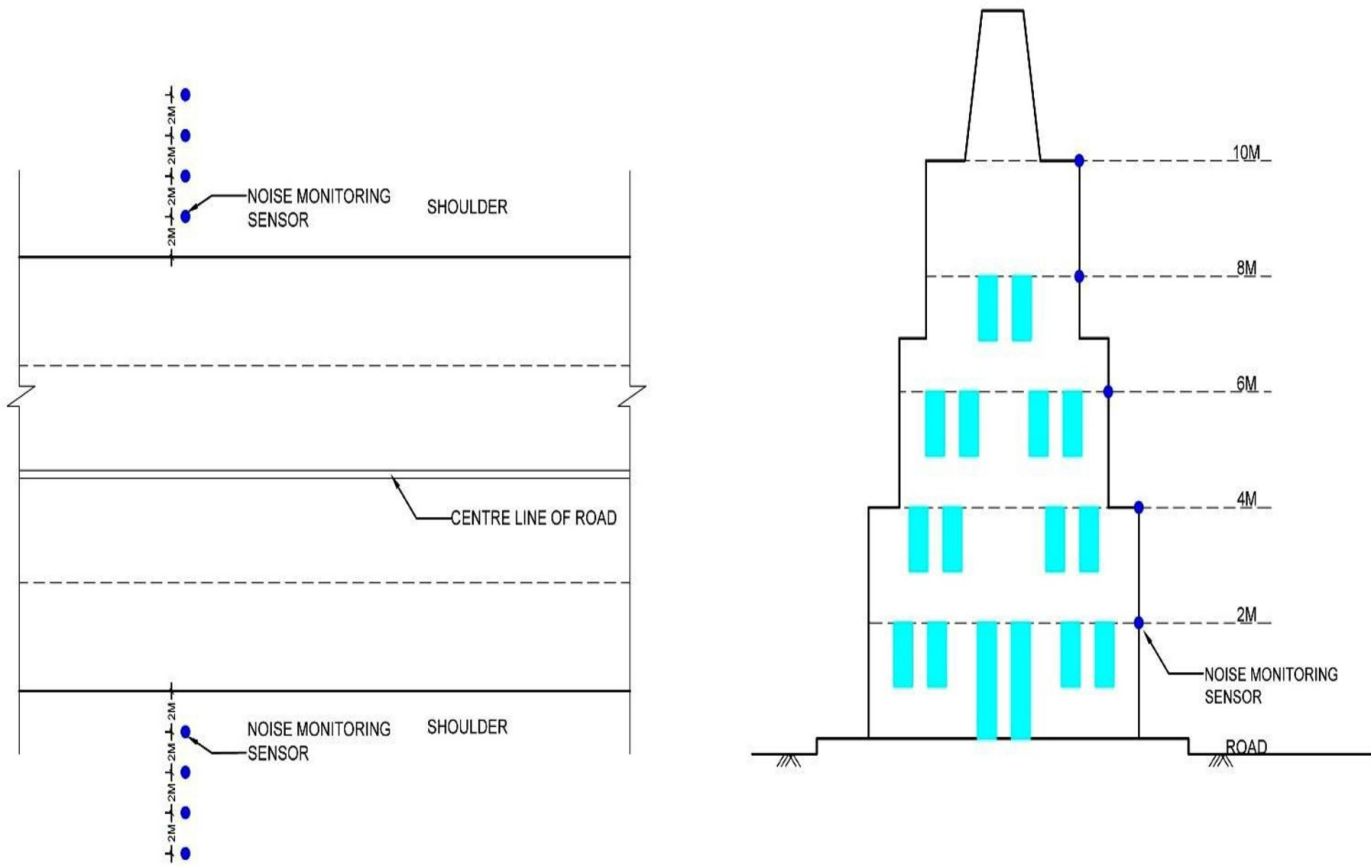
Location of noise monitoring sensors for validation of 2D and 3D noise maps.

2D and 3D noise mapping results have been compared by real-time noise monitoring at all four critical locations. As shown in the Fig 11 validation of the 3D noise map at Sabz Burj, the noise monitoring has been done at the height of 2, 4, 6, 8, and 10m. For validation of the 2D noise map, the noise monitoring has been done up to 8m at an interval of 2m from the road’s shoulder. Validation of results shown in Table 3, which indicates the maximum standard deviation for 3D noise maps remains at the height of 6m, i.e., 3.95, and maximum Standard deviation for 2D noise maps has been 2.40 at the height of 8m, which is significantly less. Data validation indicates that the 2D and 3D noise mapping using SoundPLAN (acoustic) gives very accurate results with very less standard deviation.

**Table 3:** Validation of results for 2D and 3D noise mapping.

S. No	Height/Distance	Noise Level dB (A) SoundPLAN (acoustic)	Noise Level dB (A) (Real time monitoring)	Standard Deviation	Variance
***3D Noise Mapping***					
1.	2m	72.5	73.8	0.91	0.85
2.	4m	71.5	73.4	1.34	1.81
3.	6m	65.0	70.6	3.95	15.68
4.	8m	64.3	68.8	3.18	10.13
5.	10m	62.3	65.7	2.40	5.78
***2D Noise Mapping***					
1.	2m	75.6	77.8	1.55	2.42
2.	4m	74.3	76.2	1.34	1.81
3.	6m	72.1	75.2	2.19	4.81
4.	8m	68.5	71.9	2.40	5.78

## Conclusions

In this study, noise monitoring of 13 locations of south Delhi’s residential area has been carried out. Based on noise monitoring results, critical locations have been identified. 2D and 3D noise maps have been developed for the critical locations to find out the noise exposure levels on the nearby residential buildings. The monitored results show that the selected areas of South Delhi have been continuously exposed to a very high sound pressure level. At day time, the maximum noise level is observed at Ashram Chowk [81.1 dB (A)] at 8 pm. However, the minimum noise level is found to be at Lala Lajpat Rai Marg [76.4dB (A)] at 7 pm. 2D noise maps show the variation of noise level along both sides of the roads. The 3D noise maps revealed a noise level of 55 dB (A) at the height of 12.45m, 11.39m, 10.52m, and 9.75m at Ashram Chowk, Sabz Burj, Lala Lajpat Rai Marg, and Moolchand intersection, respectively. It has also been concluded that the 2D and 3D noise mappings are instrumental approaches for understanding the noise level distribution of any area. The study indicates an urgent requirement of strategies for the improvement of the noise level. 2D and 3D noise maps are also beneficial for decision-makers to identify the critical locations that required urgent noise improvement.

## References

[1] Parka S. H., Leea J. P., and Le K. B., Levels and sources of neighbor noise in heavyweight residentialbuildings in Korea,*Applied Acoustic*, Vol. 120, pp 148–157, 2017, 10.1016/j.apacoust.2017.01.012.

[2] Bunna F., and Zannin P.H.T.,Assessment of railway noise in an urban setting,*Applied Acoustic*, Vol. 3, pp. 16–23, 2016, 10.1016/j.apacoust.2015.10.025.

[3] Ryua H., ParkbI K, Chunc B., and Chang S., Spatial statistical analysis of the effects of urban form indicators on road-traffic noise exposure of a city in South Korea,*Applied Acoustic*, Vol. 115,pp. 93–100, 2017, 10.1016/j.apacoust.2016.08.025.

[4] Shuyangb Z., Heittolab T., and Virtanen T., Environmental noise monitoring using source classification in sensors PanuMaijalaa,*Applied Acoustic*,Vol. 129, pp. 258–267, 2018, 10.1016/j.apacoust.2017.08.006.

[5] EU of Noise Policy Working Group 4 on Noise Mapping, 1999.

[6] Asakura T., Miyajima T.,and Sakamoto S., Prediction method for sound from passing vehicle transmitted through building facade,*Applied Acoustic*, Vol. 5, pp. 758–769, 2013, 10.1016/j.apacoust.2012.11.011.

[7] Cai M., Zou J., Xie J., and Ma X., Road traffic noise mapping in Guangzhou using GIS and GPS,*Applied Acoustic*, Vol. 87, pp. 94–102, 2015, 10.1016/j.apacoust.2014.06.005.

[8] Klaeboe R., Engelien E., and Steinnes M.,Context sensitive noise impact mapping,*Applied Acoustic*, Vol. 67, pp. 620–42, 2006, 10.1016/j.apacoust.2005.12.002.

[9] Stoter J., Kluijver D. H., and Kurakula V. K., 3D noise mapping in urban areas,*International Journal of Geographical InformationScience*, Vol. 22, pp. 907–924, 2008, 10.1080/13658810701739039.

[10] Gonzaleza M. D., Morillasa J. M. B., Godinhob L., and Amado M.P., Acoustic screening effect on building facades due to parking lines in urban environments. Effects in noise mapping,*Applied Acoustic*; Vol. 130, pp. 1–14, 2018, 10.1016/j.apacoust.2017.08.023.

[11] BarrigonMorillas J. M., Ortiz-Caraballo C., Prieto Gajardo C., “The temporal structure of pollution levels in developed cities,” *Science of Total Environment*; (517)3, pp. 1–7, 2015, 10.1016/j.scitotenv.2015.02.057. 25710623

[12] Akhtar N., Ahmad K., and Gangopadhyay S., Road Traffic Noise Mapping and A Case Study For Delhi Region,*International Journal of Applied Engineering and Technology*, Vol. 2 (4), pp. 39–45, 2012.

[13] John H., Andrew J., and Kevin H., The Birmingham updated noise mapping,*ActaAcustica United With Acustica*, 1:66–8, 2005.

[14] Kenneth K., Eddie D., and James C., Community and regional noise mapping in the United States,*Sound Vibration*, Vol. 9, pp. 14–7, 2007.

[15] Tsai K. T., Lin M. D., and Chen Y. H., Noise mapping in urban environments: A Taiwan study, *Applied Acoustics*, Vol. 70(7):9 pp. 64–72, 2009, 10.1016/j.apacoust.2008.11.001.

[16] Ausejo M., Recuero M.,.Asensio C. I.,Reduction in calculated uncertainty of a noise map by improving the traffic model data through two phases,*ActaAcust United Acust*.; Vol. 97:761–8, 2011, 10.3813/AAA.918456

[17] Suarez E., Barros J. L.,Traffic noise mapping of the city of Santiago de Chile,*Science of the total environment*,Vol. 466, pp. 39–46, 2014, 10.1016/j.scitotenv.2013.07.013 23948498

[18] Akhtar N., Ahmad K., and Alam P, Noise Monitoring and Mapping for Some Pre-selected Locations of New Delhi, India, *Fluctuation and Noise Letter*, Vol. 15, pp. 1–12, 2016, 10.1142/S021947751650019X.

[19] Cai M., Zou J., Xie J., and Ma X., “Road traffic noise mapping in Guangzhou using GIS and GPS” *Applied Acoustics*, Vol. 31(87), pp. 94–102, 2015, 10.1016/j.apacoust.2014.06.005.

[20] Merchan C. I., and Diaz-Balteiro L.,Noise pollution mapping approach and accuracy on landscape scales,*Science of the Total Environment*, Vol. 449:1, pp. 15–25, 2013, 10.1016/j.scitotenv.2013.01.063. 23416205

[21] Joshi A. N., Joshi N.C., Rane P. P.,Noise Mapping in Mumbai City, India, Vol. 02(3): pp. 380–385, 2015.

[22] Pinto F. A.,and Mardones, M. D.,Noise mapping of densely populated neighborhoods—example of Copacabana, Rio de Janeiro—Brazil, *Environmental monitoring and assessment*, Vol. 155(1–4):309–18, 2009, 10.1007/s10661-008-0437-9. 18663590

[23] Billah M., and Rahman G. A., “Land cover mapping of Khulna city applying remote sensing technique” In Proceedings of the 12th International Conference on Geo-informatics (Sweden: Geospatial Information Research: Bridging the Pacific and Atlantic, University of Gavle); 04; pp. 707–714, 2004.

[24] Suarez E., Barros J., Baez L., Stevens J., Romero R., and Mapa R., de Ruido de la Comuna de Santiago de Chile MedianteModelacion, International congress on acoustic and audio Profesional INGEACUS, Valdivia, Chile, 2011.

[25] Geymen A., and Bostanci B., Production of GIS aided noise maps turke, FIG working week, Rome, Italy, 2012.

[26] Gonzalez D. M., Barrigón J. M., and GozaloG R.,Theinfluence ofmicrophone location on the results of urban noise measurements, Applied Acoustic, Vol. 90, pp.64–73, 2015, 10.1016/j.apacoust.2014.11.001.

[27] Alam P., Ahmad K., and Akhtar N., Temporal and Spatial Fluctuation of Noise Levels in the Closed Vicinity of Urban Roadways, International Journal of Recent Technology and Engineering, Vol. 08, pp. 5983–5989, 2019, 10.35940/ijrte.C5914.098319.

[28] Akhtar, N., Ahmad, K., and Gangopadhyay S., Annoyance due to road traffic vehicles,” *Proceedings of Conference on Inter Noise, Lisbon, Portugal*, pp. 1–16, 2010.

[29] Protocol for ambient level noise monitoring, Central Pollution control board Delhi, pp. 1-,6 Jul 2005.

[30] ISO 1996-I: Acoustic description and measurement of environment noise Part-1: Basic quantities and procedure, 2016.

[31] JantienStoter Henk De Kluijver and VinaykumarKurakula; 3D noise mapping in urban areas; *International Journal of Geographical Information Science*; Vol. 22; pp. 907–924, 2017.

